# Associations of cognitive and behavioural impairment in ALS with brain pathology: pTDP-43 versus microglial activation

**DOI:** 10.1007/s00415-026-13848-4

**Published:** 2026-05-15

**Authors:** Hanneke M. J. Slaghekke, Rosanne Govaarts, Lucia Mesarosova, Angelika Mühlebner, Emma Beeldman, Marianne de Visser, Michael A. van Es, Leonard H. van den Berg, Anneke J. van der Kooi, Yolande A. L. Pijnenburg, Eleonora Aronica, Joost Raaphorst

**Affiliations:** 1 Department of Neurology, Medisch Spectrum Twente, Enschede, The Netherlands; 2 Department of Radiology, C.J. Gorter MRI Center, Leiden University Medical Center, Leiden, The Netherlands; 3 Department of Pathology, Amsterdam University Medical Centers, University of Amsterdam, Amsterdam, The Netherlands; 4 Department of (Neuro)Pathology, UMC Utrecht Brain Center, University Medical Center Utrecht, Utrecht, The Netherlands; 5 Department of Neurology, Rode Kruis Ziekenhuis, Beverwijk, The Netherlands; 6 Department of Neurology, Amsterdam Neuroscience, Amsterdam University Medical Centers, University of Amsterdam, Amsterdam, P.O. Box 22700 DE The Netherlands; 7 Department of Neurology, UMC Utrecht Brain Center, University Medical Center Utrecht, Utrecht, The Netherlands; 8 Alzheimer Centre, Amsterdam University Medical Centers, Vrije Universiteit, Amsterdam, The Netherlands

**Keywords:** Amyotrophic lateral sclerosis, frontotemporal dementia, cognition, behaviour, brain pathology, pTDP-43

## Abstract

**Objective:**

Investigate associations between brain pathology (pTDP-43 inclusions and microglial activation) and cognitive and behavioural impairment in patients with amyotrophic lateral sclerosis (ALS).

**Methods:**

Based on comprehensive neuropsychological examination and behavioural assessment, 21 ALS patients of whom post mortem brain tissue was obtained, were classified as having 1) no cognitive and/or behavioural impairment (pure motor ALS), 2) mild cognitive and/or behavioural impairment (ALSci/bi), and 3) ALS with behavioural variant frontotemporal dementia (ALS-bvFTD). Immunohistochemical staining of pTDP-43 and HLA-DR-defined microglial activation was semi-quantitatively assessed in grey and/or white matter of the prefrontal cortex, thalamus, hippocampus, and motor cortex.

**Results:**

Fourteen patients had pure motor ALS, four patients had ALSci/bi, and three patients had ALS-bvFTD. pTDP-43 pathology in the grey matter of the prefrontal cortex and *gyrus dentatus* differed between groups, especially between pure motor ALS and ALS-bvFTD. For each extra-motor brain region, pTDP-43 severity was highest in patients with ALS-bvFTD and lowest in patients with pure motor ALS, with ALSci/bi in between. This pattern was not observed for microglial activation. Associations between white matter pTDP-43 severity and cognitive/behavioural impairment were less robust than those in grey matter.

**Conclusion:**

Severity of cognitive and/or behavioural impairment in ALS is related to severity of pTDP-43 pathology, in particular in the grey matter of extra-motor brain regions; we did not detect a clear association with microglial activation.

## Introduction

In patients with amyotrophic lateral sclerosis (ALS), different levels of cognitive or behavioural impairment, including behavioural variant frontotemporal dementia (ALS-bvFTD), may occur [1]. Associations between cognitive and/or behavioural impairment and brain pathology in extra-motor regions of ALS patients are uncertain. There is conflicting evidence on phosphorylated(p)TDP-43 pathology in ALS with mild cognitive impairment (ALSci), and there is a lack of confirmatory studies regarding an association with microglial activation in ALSci [2–9]. Brain pathology data of patients with ALS and behavioural impairment (ALSbi) is scarce, and associations between prefrontal white matter pTDP-43 pathology with ALSci/bi or ALS-bvFTD have not been extensively studied [10], despite in vivo brain imaging studies showing white matter changes in these groups [11].

We aimed to characterize associations between cognitive and/or behavioural impairment and both pTDP-43 and microglial pathology in extra-motor brain regions in patients with ALS.

## Methods

### Participants

All patients had a diagnosis of probable or definite ALS according to the revised El Escorial Criteria and a neuropathological diagnosis of ALS [12, 13]. This study was performed in accordance with the Declaration of Helsinki and the local medical ethical committee. Written informed consent was obtained from all participants at inclusion.

### Clinical assessments

To correlate (non-motor) outcomes with respiratory and affective symptoms and measures of motor neuron degeneration, we assessed anxiety and depression, respiratory function, and the functional (motor) status of patients, using hospital anxiety and depression scale (HADS) [14], forced vital capacity (FVC), upper motor neuron (UMN) involvement [15] and, functional (motor) status (ALS functional rating scale - revised (ALSFRS-R), including slope) [16], respectively. We assessed *C9orf72* status, time of death, and presence of sepsis from clinical files.

### Neuropsychological examination

The neuropsychological examination comprised tests of executive function, language, memory functions, visuospatial functions, and social cognition, including a proxy-based behavioural questionnaire (ALS-FTD Questionnaire (ALS-FTD-Q)) [17], as described previously [18].

We used a previously reported patient categorization based on the Strong criteria [19]: 1) patients with no cognitive and/or behavioural impairment (pure motor ALS); 2) mild cognitive and/or behavioural impairment (ALSci/bi), and 3) ALS-bvFTD [20].

### Neuropathology

ALS brain tissues were collected from the Dutch ALS Tissue Bank and included prefrontal cortex (Brodmann area 10), thalamus (mediodorsal nucleus), hippocampus (*gyrus dentatus* and *Cornu Ammonis)*, and primary motor cortex. The thalamus was collected at the level of the subthalamic nucleus. In the prefrontal cortex and primary motor cortex, grey *and* white matter were studied. The brain tissue was collected according to the neuropathological protocol based on the recommendations of the BrainNet Europe consortium/BrainNet Europe Motor Neuron Disease protocol [21]. None of the cases had co-existing pathology (i.e., vascular pathology, Alzheimer’s disease, Lewy body disease, or limbic age-related TDP-43 encephalopathy).

### Immunohistochemistry

Tissue was fixed in 4% formaldehyde and routinely embedded in paraffin. Sections (5 µm) were cut with a microtome (Microm, Heidelberg, Germany) and mounted on slides (Superfrost + Menzel, Germany). Immunostainings were performed with a Ventata semiautomated staining machine (Benchmark ULTRA; Ventana, Illkirch, France) according to the manufacturer’s protocol. Antibodies used for staining are (HLA-)DP, DQ, DR for microglial activation (human leukocyte antigen class II, 1:100, clone Cr3/43, DAKO), and pTDP-43 (cosmo bio, 1:5000, clone 11.9 ps409/410). Images were acquired using an Olympus BX41 microscope with Leica IM at a magnification of 200x.

Tissue sections were evaluated by a researcher, who was blind to clinical data. A second independent evaluation was independently performed by a neuropathologist. In cases of disagreement, scores were discussed and resolved by consensus. Pathological scores for pTDP-43 were rated as 0 = absent, 1 = rare (a few pTDP-43 inclusions on a single brain section at 200 × magnification), 2 = occasional (inclusions not present in every microscopic field), 3 = moderate (a few inclusions in most microscopic fields), and 4 = numerous (many inclusions in every microscopic field) [22].

Immunoreactivity for microglia was assessed to quantify the relative number of positive cells within the lesion. The scoring was as 0 = absent (no detectable microglia), 1 = rare (few scattered microglia; 1–5 cells per 20 × field), 2 = sparse (moderate presence with dispersed distribution; 6–15 cells per 20 × field), 3 = high (numerous microglia with increased clustering; 16–30 cells per 20 × field), and 4 = very high (dense aggregation of microglia; > 30 cells per 20 × field). Intensity of staining was ranked as 0 = no staining, 1 = weak, 2 = moderate, 3 = strong staining. All areas of the lesion were examined, and the score represents the predominant cell staining intensity found in each case. The immunoreactivity score (IRS) was calculated by multiplying the immunoreactivity score by the intensity score, resulting in a value ranging from 0 (no staining) to 12 (maximum staining.[23]

### Statistical analysis

Group differences were analysed using one-way ANOVA, Kruskal–Wallis (noted as H(df)), or Fisher’s exact test. In case of significant group differences, an unpaired t-test or Mann–Whitney U test was used to analyse two groups. Z-scores of neuropsychological tests (corrected for age, gender, and educational level) were used to present a cognitive profile (group level). The behavioural profile was evaluated by reporting proportions of patients with a score > 1 on the five most frequently abnormal ALS-FTD-Q items.

In an exploratory analysis, we correlated cognitive/behavioural impairment (yes/no; ALSci/bi and ALS-bvFTD grouped) with positive pTDP-43 staining in the white matter of the prefrontal cortex, as compared to the white matter of the primary motor cortex.

All statistical tests were two-tailed. A p-value < 0.05 was considered statistically significant.

We did not perform multiple comparisons because of the exploratory nature of this study.

## Results

### Patient characteristics

Twenty-one patients were included. Fourteen patients had pure motor ALS (67%), four patients had ALSci/bi (19%), and three patients had ALS-bvFTD (14%).

Age, time between neuropsychological examination and death, FVC, HADS, ALSFRS-R, ALSFRS-R slope, and UMN-score did not differ between groups (Table 1). None of the patients had had sepsis before death. The clinical characteristics of the patients with cognitive and/or behavioural impairment are shown in Table 2.

**Table 1:** Demographic and clinical characteristics of patients

	pure motor ALS (n = 14)	ALSci/bi (n = 4)	ALS-bvFTD (n = 3)
Sex, M/F	9/5	2/2	2/1
Age (years)	63.0 (9.3)	65.9 (4.3)	65.9 (4.3)
Disease duration (months)	11.0 (7.5–24.5)	12.0 (7.5–24.5)	7.0 (6.0–9.0)
Survival (months)	32.0 (18.8–50.8)	27.0 (15.8–66.0)	15.0 (11.0–26.0)
Time between neuropsychological examination and death (months)^	16.5 (3.3–22.8)	5.0 (1.3–18.5)	5.0 (4.0–13.0)
FVC %	86.1 (60.9–100.2)	80.3 (50.7–103.9)	85.1 (79.2–94.0)
HADS	7.4 (3.8–11.3)	5.25 (0.8–11.5)	5.0 (1.0–10.0)
Premorbid IQ	114.5 (100.8–123.8)	96.5 (65.5–118.5)	115.0 (94.0–119.0)
C9orf72 mutation (no.)^#^	0	2	1
Site of onset, B/L/T/R	6/7/1/0	1/3/0/0	0/3/0/0
Bulbar involvement (no)	13	4	1
ALSFRS-R	33.5 (26.5–38.5)	33.5 (23.8–41.0)	40.0 (31.0–44.0)
ALSFRS-R slope	0.90 (0.75–1.65)	0.86 (0.34–3.60)	1.14 (0.44–2.83)
UMN-score	24.0 (16.0–30.5)^##^	26.0 (18.0–31.8)^##^	30.0 (21.0–32.0)

Data are shown as mean (standard deviation) or median (interquartile range) when appropriate. None of the variables showed a statistically significant difference between groups. Survival is the time between disease onset and death. FVC% is the percentage of the predicted value of the forced vital capacity. ALSFRS-R is a 12-item scale with a maximum of 48, which indicates no physical impairment. The ALSFRS-R slope is calculated as 48—ALSFRS-R score at the time of the neuropsychological examination divided by the number of months between first symptoms and the neuropsychological examination. The UMN-score is a scale for the involvement of the central (upper) motor neuron, with a normal score of 16 (range 0–48); *ALS* amyotrophic lateral sclerosis, *ALSci*/*bi* ALS with cognitive and/or behavioural impairment, *ALS*-*bvFTD* ALS with behavioural variant frontotemporal dementia, *FVC* forced vital capacity, *HADS* hospital anxiety and depression scale, *no *number, *B* bulbar onset, *L* limb onset, *T* truncal onset, *R* respiratory onset, *ALSFRS*-*R* amyotrophic lateral sclerosis functional rating scale-revised; ^none of the patients underwent tracheostomy; #one patient (ALS-bvFTD) had familial ALS (C9orf72 positive), all other patients had sporadic ALS. ##Two patients (one patient with pure motor ALS, another patient with ALS-bvFTD) initially presented with progressive muscular atrophy, which progressed to ALS

**Table 2:** Clinical characteristics of ALS patients with cognitive and/or behavioural impairment

Case (no.)	Sex	Cognitive impairment	Behavioural impairment	ALSFRS-R slope	UMN score	Time NPE-death^#^
1	F	None	bvFTD	2.83	30	4
2	M	Mild	bvFTD	1.14	21	13
3	M	Severe	bvFTD	0.44	32	5
4	M	None	Mild	0.18	33	5
5	M	Severe	Mild	4.50	24	0
6	F	Mild	None	0.83	28	23
7	F	Mild	None	0.89	16^##^	5

ALSFRS-R slope = the decrease in ALSFRS-R per month, calculated as 48—ALSFRS-R score at time of the NPE divided by the number of months between first symptoms and the NPE; *UMN* upper motor neuron, scale for involvement of the upper motor neuron, with a normal score of 16 (range 0–48); *NPE* neuropsychological examination, *bvFTD* behavioural variant of frontotemporal dementia. #Duration between NPE and death in months. ##This patient initially presented with progressive spinal muscular atrophy, which developed into ALS

### Neuropsychological examination

**Fig. 1:**
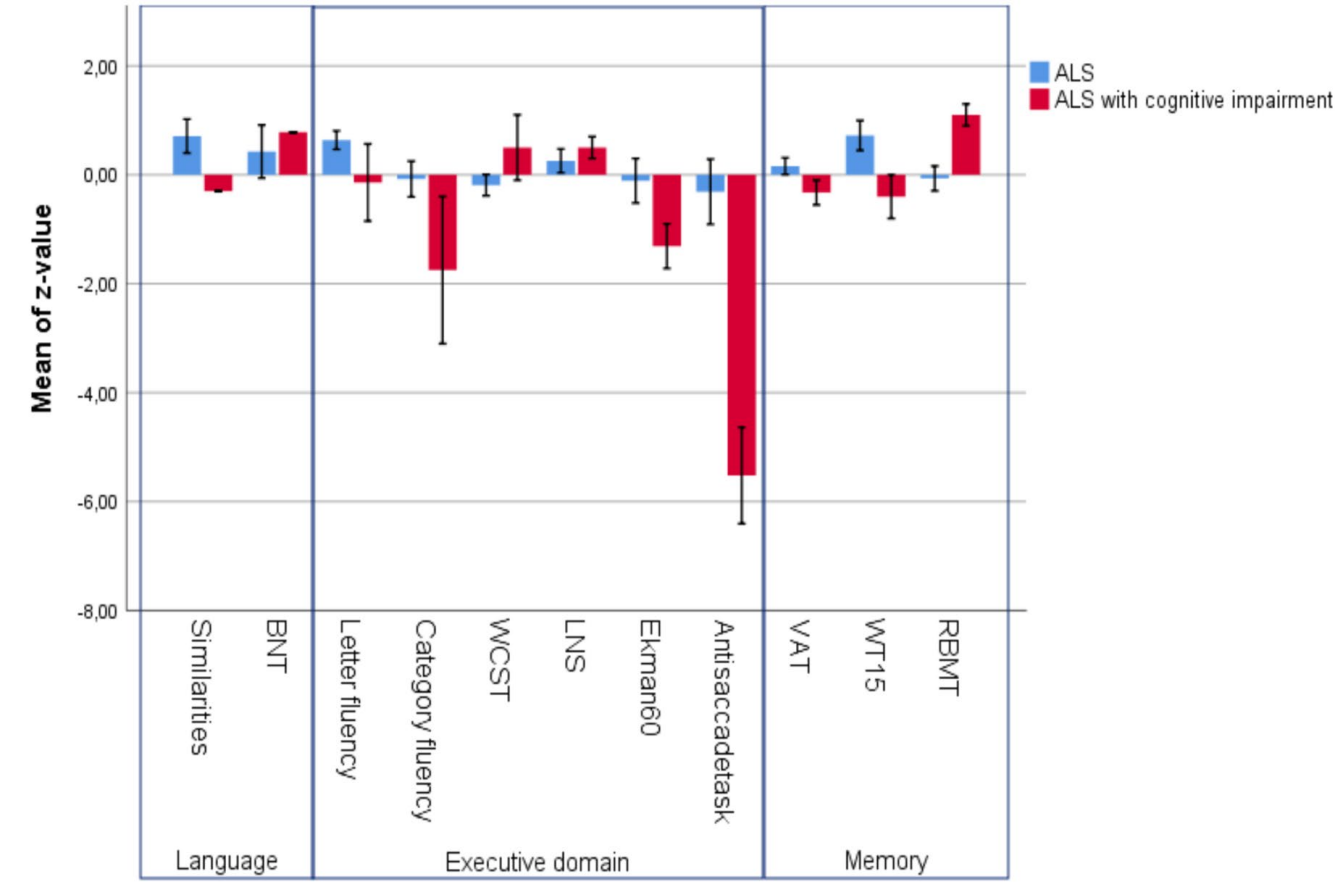
Performance on neuropsychological tests. Data of all the patients are shown. Red bars indicate the performance of the group of patients with cognitive impairment (ALSci; cases #2, #3, #5, #6, and #7). Error-bars display standard error of the mean. *ALS* amyotrophic lateral sclerosis, *BNT* Boston naming test, *WCST* Wisconsin card sorting test, *LNS* letter number sequencing, *VAT* visual association test, *WT15 *15-word learning test; *RBMT* Rivermead behavioural memory test

Cognitive impairments were found in the executive domain, i.e. category fluency (pure motor ALS z = 0.043 (1.12); ALSci z = − 1.78 (1.47)), anti-saccade test (pure motor ALS z = −0.30 (0.69); ALSci z = − 6.63 (2.92)), social cognition domain (Ekman 60 faces (pure motor ALS z = − 0.61 (1.37); ALSci z = − 3.08 (3.15)) and to a lesser extent in the language domain. The cognitive profile is shown in Fig. 1. The mean sum of differences was 1.03 (3.59) in patients with pure motor ALS and −10.16 (8.05) in patients with ALSci.

The behavioural profile of patients with ALS-bvFTD and ALSbi was characterized by a loss of interest/apathy (in 4 out of 5 patients (4/5)), irritability (4/5), restlessness (5/5), emotional changes/instability (5/5), and poor judgment/assessment of situations (5/5).

### pTDP-43 inclusions and microglial activation

**Fig. 2:**
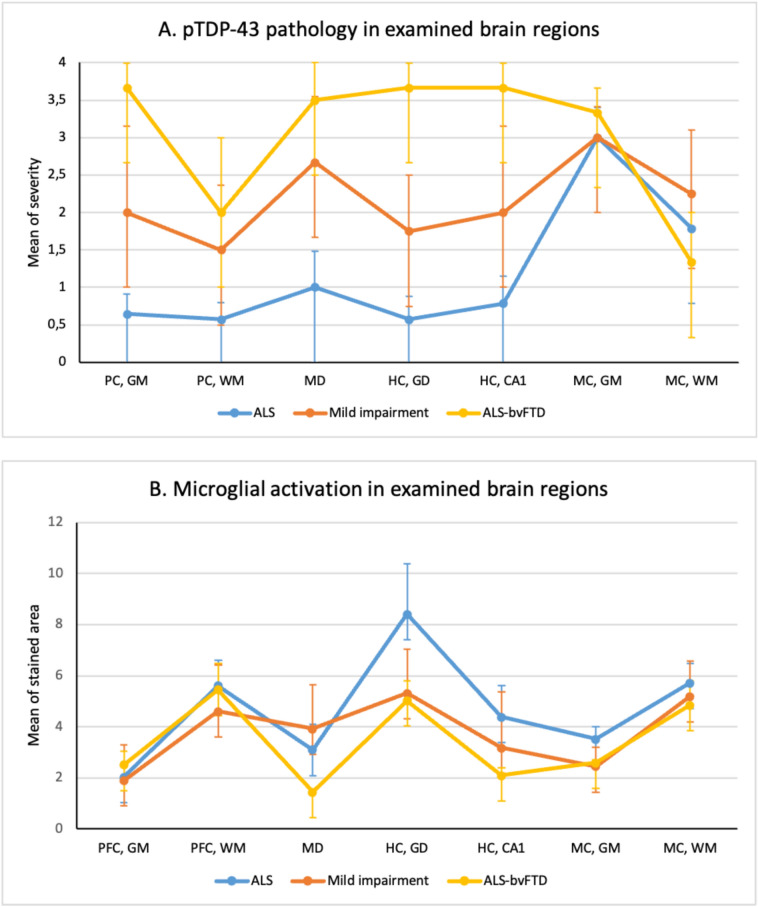
pTDP-43 pathology and microglial activation in brain regions of ALS patients. Panel A shows the severity of pTDP-43 pathology, and panel B shows the presence of microglial activation with HLA-DR staining in different brain regions of patients with ALS. Data are presented as mean and standard error. The connecting lines between regions enhance the readability of the figure. *PFC* prefrontal cortex, *GM* grey matter, *WM* white matter, *MD* mediodorsal nucleus of thalamus, *HC* hippocampus, *GD* gyrus dentatus, *MC* motor cortex, *ALS* amyotrophic lateral sclerosis, *ALSci*/*bi* ALS with cognitive and/or behavioural impairment, *ALS-bvFTD* ALS with behavioural variant frontotemporal dementia, *IRS* immunoreactivity score

pTDP-43 scores in the grey matter of the prefrontal cortex (H(3) = 8.227, p = 0.042) and in the *gyrus dentatus* (H(3) = 11.026, p = 0.012) differed between pure motor ALS, ALSci/bi, and ALS-bvFTD (Fig. 2A). pTDP-43 scores differed in the grey matter of the prefrontal cortex and the *gyrus dentatus* between pure motor ALS and ALS-bvFTD (Prefrontal cortex: U = 41.5, n = 17, p = 0.003; *gyrus dentatus*: U = 41.0, n = 17, p = 0.006). For all brain regions except the motor cortex, the severity of pTDP-43 inclusions was highest in ALS-bvFTD, lowest in pure motor ALS, with ALSci/bi in between (Fig. 2A).

Five out of 14 patients with pure motor ALS (36%; median time between neuropsychological examination and death was 13 months (range 5–18)) had mild (n = 4) to severe (n = 1) pTDP-43 pathology in one or more extra-motor regions.

Microglial activation (HLA-DR) was present, to some extent, in all patients, in all brain regions, and did not differ between groups (Fig. 2B).

**Fig. 3:**
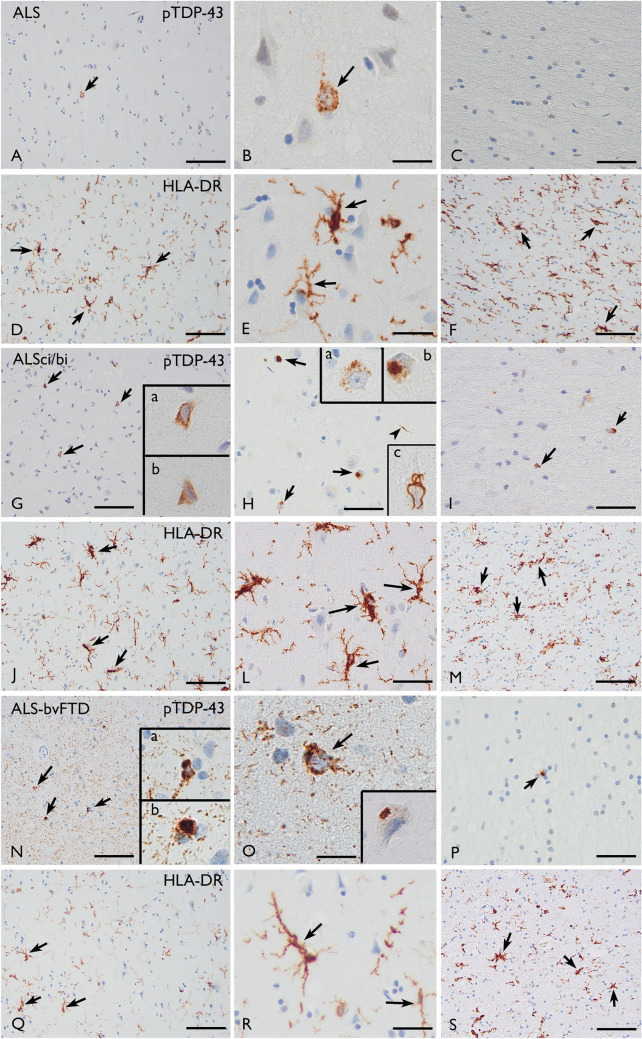
pTDP43 pathology and HLA-DR expression in the prefrontal cortex. Panels A-F (pure motor ALS). A–C: Representative photomicrographs of pTDP-43 immunoreactivity in grey matter (A–B) and white matter (C). Arrows in A–B indicate pTDP-43-positive neurons. D–F: Representative photomicrographs of HLA-DR immunoreactivity in grey matter (D–E) and white matter (F). Arrows highlight HLA-DR-positive microglial cells. Panels G-M (ALSci/bi). G–I: Representative photomicrographs of pTDP-43 immunoreactivity in grey matter (G–H) and white matter (I). Arrows in G–H indicate pTDP-43-positive neurons; occasional positivity in neurites (arrowhead in H). High-magnification views of neurons with intraneuronal inclusions are shown in insets a–b in G and a–c in H; arrows in I indicate positivity in glial cells. J-M: Representative photomicrographs of HLA-DR immunoreactivity in grey matter (J-L) and white matter (M). Arrows highlight HLA-DR-positive microglial cells. Panels N-S (ALS-bvFTD). N–O: Representative photomicrographs of pTDP-43 immunoreactivity in grey matter (N–O) and P in white matter. Strong neuropil positivity for pTDP-43 is observed. Arrows in N–O indicate pTDP-43-positive neurons. High-magnification views of neurons with intraneuronal inclusions are shown in the insets of G and H. Arrows in P indicate pTDP-43-positive glial cells. Q–S: Representative photomicrographs of HLA-DR immunoreactivity in grey matter (Q–R) and white matter (S). Arrows highlight HLA-DR-positive microglial cells. Scale bars: A, D, F, G, J, M, N, Q, S: 80 µm. B, C, E, H, I, L, O, P, R: 40 µm

Representative photographs of pTDP-43 and HLA-DR expression in the prefrontal cortex are shown in Fig. 3.

Regarding the white matter, pTDP-43 positive inclusions were found in 9 patients (43%) in the prefrontal cortex, more severe in ALSci/bi (n = 4), as compared to pure motor ALS (n = 5; t = 6,857, p = 0.009, df = 1). The number of pTDP-43 positive inclusions in the white matter of the primary motor cortex was similar between the groups.

## Discussion

Our study indicates that the degree of cognitive and behavioural impairment in ALS is associated with pTDP-43 pathology in the grey matter of the prefrontal cortex and the dentate gyrus of the hippocampus; we did not detect a clear association with HLA-DR-defined microglial activation.

To our knowledge, one other post-mortem study in ALS used a similarly comprehensive neuropsychological examination to characterize the cognitive profile. However, the authors reported no difference between pTDP-43 pathology in patients with and without cognitive impairment [5]. Although patient numbers in both studies are too small for robust conclusions, the addition of assessment of behavioural impairment to our definition (ALSci/bi) may have contributed to the association between pTDP-43 pathology in frontotemporal brain regions and different levels of severity of the cognitive/behavioural symptoms in ALS. Our findings are in agreement with others, who reported associations between frontotemporal pTDP-43 pathology with ALSci – as defined by a restricted number of cognitive tests – and with ALS-bvFTD or ALS-dementia [4, 6, 8, 9].

Some patients with the clinical diagnosis of pure motor ALS had mild to severe pTDP-43 pathology in extra-motor regions. Apart from cell protective mechanisms delaying cognitive impairment [24], another explanation might be the development of brain pathology between the neuropsychological and post-mortem examination (median 5 months, range 0–23 months). The latter is supported by findings of cognitive and behavioural deterioration in a quarter of previously pure motor ALS patients [18].

Our study may contribute to the ongoing debate on the relevance of microglial activation in extra-motor regions in ALS [25, 26]. In our study, no clear association was detected between HLA-DR-defined microglial activation and ALSci/bi, which is in agreement with previous observations [2], but contrasts with findings by others in patients with ALSci (or isolated executive impairment) [4]. However, comparisons between studies are somewhat hampered by different markers for microglial activation. Future studies may unravel whether activated microglia, as found in extra-motor regions using positron emission tomography (PET), are an epiphenomenon or significantly contribute to the disease process related to ALSci/bi [25, 27].

Our finding of an association between white matter pTDP-43 pathology in the prefrontal cortex and ALSci/bi may be considered hypothesis-generating. This association has been shown in patients with FTD without ALS and may reflect white matter microstructural changes, which were also found on brain imaging studies of ALS patients [10, 11].

In addition to its strengths (post-mortem study with well characterised patients along the ALS-bvFTD disease spectrum), our study also has limitations: the small sample size limits statistical power, and we cannot sufficiently rule out a type 2 error regarding the microglial findings. We did not include healthy controls to match the microglial activation. We have previously shown that immunocytochemistry with antibodies against the major histocompatibility complex (MHC) class II-antigens (HLA-DR) showed only scattered immunoreactive cells in control autopsy brains [28, 29]. Also, we did not assess the morphology of the microglia, because we felt this was beyond the scope of this exploratory study. Another important limitation is the significant time lag between neuropsychological evaluation and death in some of our patients (median 5 months; maximum 23 months), which may hamper the interpretation of the clinical-pathological correlations of this study. Further, we did not subject all our patients to genetic testing, so we could not analyse to what extent the results would be C9orf72-driven.

In conclusion, our study in ALS suggests an association between cognitive and/or behavioural impairment and extra-motor region pTDP-43 pathology; we did not detect a clear association with HLA-DR-defined microglial activity. These findings contribute to the understanding of the underlying pathological signature of the ALS-bvFTD disease continuum.

## Data Availability

The datasets used and analysed in this study are available from the corresponding author on reasonable request.
